# Differences in Hemodialysis Claim Patterns Across Membership Types Among Patients With Renal Failure Based on National Health Insurance Data From 2017 to 2022: Cross-Sectional Analysis

**DOI:** 10.2196/73731

**Published:** 2025-11-03

**Authors:** Aries Munandar, Syarif R Hasibuan, Dian Kusuma

**Affiliations:** 1 Department of Social Welfare Faculty of Social and Political Science University of Bengkulu Bengkulu Indonesia; 2 Faculty of Medicine Universitas Pembangunan Nasional Veteran Jakarta Jakarta Indonesia; 3 Center for Health Administration and Policy Studies Faculty of Public Health University of Indonesia Depok Indonesia; 4 Department of Public Health and Epidemiology College of Medicine and Health Sciences Khalifa University of Science and Technology Abu Dhabi United Arab Emirates

**Keywords:** hemodialysis, health equity, kidney failure, BPJS Kesehatan, Indonesia, national health insurance, claims data

## Abstract

**Background:**

Chronic kidney disease and end-stage renal disease are major contributors to the disease burden in low- and middle-income countries, including Indonesia. Despite the expansion of universal health coverage through Badan Penyelenggara Jaminan Sosial (BPJS) Kesehatan, Indonesia’s national health insurance program, disparities in access to hemodialysis persist across different socioeconomic and geographic groups. Understanding these inequities is critical to advancing equitable health care access.

**Objective:**

This study aimed to examine disparities in hemodialysis claim patterns as a proxy for access among adult patients with renal failure enrolled in BPJS, focusing on differences by membership type, sex, age, geographic region, urbanicity, and facility ownership.

**Methods:**

We conducted a cross-sectional analysis of 38,383 anonymized health insurance claims between 2017 and 2022 for patients with renal failure who were aged ≥18 years. The primary outcome was receipt of hemodialysis. We used multivariate logistic regression to estimate adjusted odds ratios (aORs) for receiving hemodialysis across BPJS membership types and other covariates. Subgroup analyses were performed by sex, facility ownership, urbanicity, and geographic region. Robust SEs and probability weights were applied to account for the sample design.

**Results:**

Of the total renal failure claims, 75.6% (29,017/38,383) involved hemodialysis. Compared with individuals in the lowest income group (ie, members subsidized under the national government budget), informal workers (aOR 1.56, 95% CI: 1.34-1.82); *P*<.001) and members subsidized under the local government budget (aOR 1.31, 95% CI: 1.05-1.63); *P*=.017) had higher odds of receiving hemodialysis, while formal sector workers had lower odds (aOR 0.81, 95% CI: 0.68-0.98); *P*=.028). Disparities were more pronounced in rural areas and among women; for example, in rural regions, locally subsidized members had more than twice the odds of receiving hemodialysis compared with nationally subsidized members (aOR 2.40, 95% CI: 1.78-3.23). Men had higher odds than women (aOR 1.17, 95% CI: 1.04-1.32), and younger patients were more likely to receive treatment than older ones. Regional disparities were stark, with patients in Java or Bali having much greater access (aOR 8.30, 95% CI 5.33-12.94) compared with those in eastern Indonesia (Papua, Maluku, and Nusa Tenggara). Patients treated at private facilities (aOR 1.30, 95% CI 1.13-1.50) and in outpatient settings (aOR 3.74, 95% CI 3.36-4.17) were more likely to receive hemodialysis, whereas those in lower-level hospitals or clinics were less likely to access care.

**Conclusions:**

Substantial disparities in hemodialysis claim patterns (as a proxy for access) exist within Indonesia’s national health insurance system, particularly affecting low-income populations, rural residents, women, and those in less advantaged regions. Policy efforts to enhance health infrastructure, improve service distribution, and reduce geographic and socioeconomic barriers are urgently needed to support equitable access to renal care services and achieve universal health coverage goals.

## Introduction

Chronic kidney disease (CKD) and end-stage renal disease (ESRD) are growing public health challenges globally, with over 800 million individuals affected and 1.5 million deaths reported in 2021, making CKD the 11th leading cause of mortality worldwide [1,2]. ESRD, the most severe form of CKD, requires renal replacement therapy in the form of hemodialysis, peritoneal dialysis, or kidney transplantation to sustain life. In low- and middle-income countries (LMICs) like Indonesia, the burden of CKD is accelerating due to aging populations and rising prevalence of 2 major risk factors—diabetes and hypertension [3]. In 2021, CKD caused more than 51,000 deaths and nearly 2 million disability-adjusted life years in Indonesia alone, underscoring its substantial contribution to the national disease burden [4].

Renal replacement therapy, particularly hemodialysis, has become a crucial component of Indonesia’s response to the CKD burden. While peritoneal dialysis and transplantation are also clinically viable, hemodialysis remains the dominant modality in Indonesia due to limited infrastructure, specialist training, and system familiarity with alternative options [5,6]. In financing this care, Indonesia’s national health insurance program, Jaminan Kesehatan Nasional, launched in 2014 to advance universal health coverage, plays a central role. Administered by the national health insurance agency (in Indonesian: the Badan Penyelenggara Jaminan Sosial [BPJS]), the program operates as the sole public payer, overseeing reimbursements for hospital claims and managing health care financing nationwide. As of May 2025, BPJS covered over 280 million members, making it one of the largest single-payer health insurance schemes globally. BPJS pools funds from government subsidies, payroll contributions, and self-paid members to deliver near-universal health coverage through both public and private providers [7,8]. In 2023, alone, it financed nearly 1.5 million renal failure claims, with hemodialysis treatment costs reaching Rp 2.91 trillion (approximately US $180 million) [9].

BPJS members fall into 2 broad categories: subsidized and nonsubsidized. Subsidized members include individuals with low and near-low income, whose premiums are paid by the national government or by local governments. Nonsubsidized members pay their own premiums or have them covered by employers and include informal workers, formal sector employees, and others such as pensioners and veterans [10,11]. Despite expanded coverage, previous studies suggest that subsidized members underuse hospital-based services compared to wealthier, nonsubsidized groups [7,8]. This raises questions about whether universal health coverage alone is sufficient to ensure equitable access to high-cost, life-sustaining therapies like dialysis [12].

Globally, LMICs have struggled to achieve equitable access to dialysis due to geographic, financial, and systemic constraints [13]. In countries like India, Nigeria, and Kenya, dialysis access is shaped by regional disparities in facility availability, infrastructure, and human resources [14]. In Indonesia, patients in eastern provinces and rural areas often face long travel distances, limited provider capacity, and inconsistent referral pathways [6,15,16]. Disparities may also arise from Indonesia’s dual public-private provider system, where private facilities—often concentrated in urban areas—may offer greater availability or faster access to hemodialysis services [12].

Existing research on ESRD inequalities has largely come from high-income countries such as South Korea and Canada, where administrative claims data are more comprehensive and health system structures differ significantly from those in LMICs [17,18]. Studies in LMICs often rely on alternative data sources such as national health surveys, which, while informative, are susceptible to issues like recall bias [19-21]. While some studies in Indonesia have used national claims data to examine disparities in health care use—such as for cardiovascular diseases, diabetes management, HIV or AIDS, and catastrophic health expenditures—there has been little focus on disparities in access to dialysis services [7,8,10,22]. These gaps in the literature limit our understanding of how systemic and socioeconomic factors shape access to life-saving treatments like dialysis in LMIC settings.

To address this critical evidence gap, we analyze national health insurance claims from 2017 to 2022 to examine disparities in hemodialysis claim patterns as a proxy for access among adult patients with renal failure in Indonesia. This study investigates differences across BPJS membership types, sex, age, urban versus rural residence, geographic region, and health facility characteristics. In the context of near-universal coverage under BPJS, disparities in claims can reasonably be interpreted as reflecting disparities in access, while acknowledging that claims represent episodes of care rather than individual patient trajectories [11,23]. We hypothesize that subsidized members, rural residents, and patients in less advantaged regions (eg, eastern Indonesia) face significantly lower odds of receiving hemodialysis compared with their nonsubsidized, urban, and Java- or Bali-based counterparts. Understanding these disparities is essential to inform evidence-based policy reforms that promote equitable access to essential health services and strengthen Indonesia’s progress toward universal health coverage.

## Methods

### Study Design

We conducted a cross-sectional observational analysis using administrative claims data for adult patients (aged ≥18 years) diagnosed with renal failure between January 1, 2017, and December 31, 2022. Renal failure cases were identified based on *International Classification of Diseases-10 (ICD-10)* codes N17 to N19, which capture acute kidney injury, CKD, and unspecified kidney failure diagnoses [24].

### Data

This study used deidentified administrative claims data from Indonesia’s national health insurance agency (BPJS), drawn from a standardized 1% random sample of insured members. The dataset includes inpatient and outpatient claims from secondary and tertiary referral health facilities between 2017 and 2022. BPJS insures over 248 million individuals; in 2022 alone, the 1% sample represented approximately 2.4 million individuals selected through simple random sampling at the household level. These members generated more than 3.3 million primary care visits and 1.2 million inpatient claims [7,8].

For this study, we extracted noncapitation claims and identified renal failure cases using *ICD-10* codes N17 to N19. After excluding individuals aged <18 years and claims without relevant diagnoses, the final analytic sample consisted of 38,383 claims, representing 7916 unique patients. A detailed flowchart outlining the sample selection process is provided in Multimedia Appendix 1.

Patient counts varied by year, from 974 in 2017 to 1589 in 2022 (Multimedia Appendix 2). However, only a small proportion of patients had repeated claims across years, for example, just 30.7% of patients from 2017 appeared again in 2018—limiting the potential for longitudinal (panel) analysis. Given this limited overlap, we adopted a cross-sectional, claim-level analytic approach, which allowed us to preserve the clinical detail of each hospitalization episode and to reflect changes in BPJS membership status within the year and over time.

Although the dataset was not stratified by diagnosis or geography, sampling probability weights were applied in all descriptive and multivariate analyses to adjust for unequal selection probabilities and enhance national representativeness. This sampling approach is aligned with international standards, such as the National Sample Cohort from South Korea’s National Health Insurance Service, and enables robust estimation of disparities in health care access and outcomes within Indonesia’s national insurance system [10,11].

### Variables

The primary outcome variable in this study was receipt of hemodialysis, defined as a binary variable (1=received hemodialysis; 0=did not receive hemodialysis). Procedure information in the dataset is coded using *ICD-9-CM* codes, including hemodialysis (n=3995), peritoneal dialysis (n=5498), and kidney transplantation (n=5569). In the final analytic sample, 75.6% (29,017/38,383) of claims between 2017 and 2022 involved hemodialysis. In contrast, claims involving peritoneal dialysis or transplantation accounted for only a very small proportion of claims.

The key independent variable in this study was BPJS membership type, categorized into 5 official groups. The first group, nationally subsidized members, represents individuals in the lowest income group as defined by the national standard through Indonesia’s Unified Database for Social Welfare (DTKS), whose premiums are fully subsidized by the central government. The second group, locally subsidized members, includes members with near-low income subsidized by local government budgets and identified according to local eligibility criteria. Nonsubsidized members comprised 3 categories: informal nonworkers, which includes employers, investors, pensioners, and veterans; informal workers, encompassing informal workers who make direct contributions to BPJS; and formal sector employees, representing formal workers whose contributions are jointly funded by employees and employers. This classification is consistent with approaches used in recently published studies [7,11,23].

Additional covariates in this study included the year of the claim, sex, age (calculated by subtracting the date of birth from the date of visit), type of residence (urban for city areas and rural for regency or district), and geographic region, categorized into Java or Bali, Sumatra, Sulawesi, Kalimantan, and the eastern region (including Papua, Maluku, and Nusa Tenggara). Facility-level characteristics were also considered, encompassing service type (inpatient or outpatient), facility ownership (government or private), facility type (hospital or specialist clinic), and facility level (classified as hospital levels A, B, C, D, or other). Clinics are referral-level health care facilities staffed by specialists and equipped to provide hemodialysis treatment. These hospital classifications reflect the service capacity and role within the health system: class A hospitals are national referral centers with the highest service complexity, advanced technologies, and full subspecialty care; class B hospitals serve as referral centers at the provincial or large district level; class C hospitals offer general inpatient services with more basic specialty care; and class D hospitals provide limited inpatient services. The “other” category includes specialized facilities such as military hospitals and hospitals focused on specific services (eg, dialysis centers, surgical centers, and pulmonary hospitals).

### Data Analysis

Multivariate logistic regression analyses were conducted to examine associations, with results expressed as adjusted odds ratios (aORs). The primary findings were derived from these multivariate models. Analyses were performed for the full sample and stratified by subgroups, including sex, facility ownership, urbanicity, and geographic region (Java or Bali and non-Java or Bali). To account for the complex sampling design, robust standard errors with probability weights were applied using the *pweight* function of Stata (version 15.1; StataCorp LLC), allowing for population-level inferences. All statistical analyses were conducted in Stata with statistical significance defined as a *P* value of .05 or lower.

### Ethical Considerations

This study involved a secondary analysis of deidentified, publicly available administrative health insurance claims data from Indonesia’s national health insurance system (BPJS). The dataset contains no personal identifiers and is not linked to individual identities, ensuring the privacy and confidentiality of all individuals represented. The study protocol was reviewed and approved by the Research and Community Engagement Ethical Committee at the Faculty of Public Health, Universitas Indonesia (573/UN2.F10.D11/PPM.00.02/2024). Informed consent is not applicable because this study used secondary data that were deidentified and publicly available. In terms of privacy and confidentiality, all data were fully anonymized, and no individual-level identifiers were accessible to the research team.

## Results

### Renal Failure Claims by BPJS Membership Type and Other Characteristics

Table 1 highlights the distribution of national health insurance claims for BPJS members in Indonesia diagnosed with renal failure between 2017 and 2022. Among the 38,383 claims recorded for renal failure, 29,017 (75.6%) claims were attributed to hemodialysis treatment. When categorized by membership type, informal workers and formal workers accounted for most total claims, with 19,064 (49.7%) claims and 9180 (23.9%) claims, respectively. A similar pattern emerged for hemodialysis claims, where informal workers and formal sector employees represented 50.5% (14,640/29,017) and 24.3% (7,041/29,017) of claims, respectively. In contrast, nationally subsidized members and locally subsidized members contributed to a smaller proportion, with 3284 (11.3%) and 1988 (6.9%) claims, respectively. Informal nonworkers accounted for 2064 (7.1%) of the claims involving hemodialysis.

Demographic and regional variables offer further insights into the distribution of claims. In terms of sex, men accounted for a greater proportion of total renal failure claims than women, with 57.1% (21,908/38,383) compared with 42.9% (16,475/38,383). Age-wise, claims for renal failure increased with age, ranging from 18.1% (6,933/38,383) among individuals aged 18 to 39 years to 30.1% (11,548/38,383) among those aged ≥60 years. Regarding urbanicity, urban residents had a marginally higher share of claims (20,188/38,383, 52.6%) than rural residents (18,195/38,383, 47.4%). Regionally, most claims (24,410/38,383, 63.6%) originated from the densely populated and more advantaged regions of Java and Bali, followed by 26.3% (10,089/38,383) from Sumatra. In contrast, Sulawesi accounted for only 2.7% (1,027/38,383) of claims, while Papua, Maluku, and Nusa Tenggara contributed just 1.8% (673/38,383).

Facility-level characteristics also shed light on the distribution of renal failure claims. By service type, outpatient services accounted for most claims (28,181/38,383, 73.4%), while inpatient services comprised 26.6% (10,202/38,383). Regarding facility type, the vast majority of claims (34,925/38,383, 91%) were associated with hospitals, with only 9% (3,458/38,383) originating from clinics. When examined by facility ownership, government-owned health facilities handled 56.2% (21,555/38,383) of claims, compared with 43.8% (16,828/38,383) managed by private facilities. In terms of hospital classification, level B hospitals recorded the highest share of total claims (14,433/38,383, 37.6%), followed closely by level C hospitals (12,543/38,383, 32.7%).

**Table 1:** Descriptive statistics of renal failure claims among adult members of Badan Penyelenggara Jaminan Sosial (BPJS) Kesehatan, Indonesia’s national health insurance program, between 2017 and 2022 (N=38,383).

Variable		Total, aOR (95% CI)	Male (n=21,908), aOR (95% CI)	Female (n=16,475), aOR (95% CI)	Government (n=16,828), aOR (95% CI)	Private (n=21,555), aOR (95% CI)
**Membership**						
	Nationally subsidized	Ref	Ref	Ref	Ref	Ref
	Locally subsidized	1.31^a^ (1.05-1.63)	1.72^b^ (1.29-2.29)	1.24 (0.89-1.72)	1.76^b^ (1.34-2.31)	1.48^a^ (1.06-2.06)
	Informal nonworker	1.07 (0.87-1.32)	0.99 (0.72-1.36)	1.34 (0.98-1.85)	0.72 (0.52-1.01)	1.62^b^ (1.22-2.16)
	Informal worker	1.56^b^ (1.34-1.82)	1.27^a^ (1.05-1.53)	2.51^b^ (1.94-3.23)	1.21 (1.00-1.48)	2.59^b^ (2.06-3.27)
	Formal worker	0.81^a^ (0.68-0.98)	0.79 (0.62-1.01)	1.06 (0.78-1.45)	0.81 (0.62-1.04)	1.18 (0.90-1.55)
**Data year**						
	2017	Ref	Ref	Ref	Ref	Ref
	2018	0.80^b^ (0.67-0.94)	0.72^b^ (0.58-0.90)	0.82 (0.64-1.06)	0.91 (0.72-1.16)	0.71^a^ (0.54-0.92)
	2019	0.83^a^ (0.71-0.98)	0.69^b^ (0.56-0.87)	0.89 (0.69-1.16)	0.93 (0.73-1.19)	0.78^a^ (0.60-1.00)
	2020	0.81^a^ (0.67-0.97)	0.82 (0.65-1.04)	0.72^a^ (0.54-0.95)	0.66^b^ (0.48-0.90)	0.83 (0.64-1.08)
	2021	0.76^b^ (0.63-0.91)	0.76^a^ (0.60-0.98)	0.74^a^ (0.55-0.99)	0.87 (0.66-1.14)	0.62^b^ (0.46-0.82)
	2022	0.97 (0.81-1.16)	1.07 (0.85-1.35)	0.92 (0.70-1.22)	0.98 (0.77-1.25)	0.99 (0.74-1.31)
**Sex**						
	Female	Ref	Ref	Ref	Ref	Ref
	Male	1.17^b^ (1.04-1.32)	—^c^	—	1.38^b^ (1.14-1.66)	1.24^b^ (1.07-1.45)
**Age group (years)**						
	18-39	Ref	Ref	Ref	Ref	Ref
	40-49	1.37^b^ (1.16-1.63)	1.69^b^ (1.31-2.17)	1.10 (0.88-1.37)	1.35^a^ (1.04-1.75)	1.26 (0.99-1.59)
	50-59	0.90 (0.76-1.07)	1.45^b^ (1.17-1.80)	0.58^b^ (0.45-0.74)	1.08 (0.86-1.38)	0.54^b^ (0.43-0.68)
	≥60	0.74^b^ (0.63-0.86)	0.97 (0.77-1.21)	0.54^b^ (0.43-0.68)	0.83 (0.65-1.04)	0.62^b^ (0.50-0.77)
**Urbanicity**						
	Rural	Ref	Ref	Ref	Ref	Ref
	Urban	1.07 (0.92-1.24)	1.04 (0.86-1.27)	1.08 (0.85-1.38)	1.42^b^ (1.17-1.72)	0.73^b^ (0.58-0.91)
**Region**						
	Papua, Maluku, NT^d^	Ref	Ref	Ref	Ref	Ref
	Java, Bali	8.30^b^ (5.33-12.94)	12.50^b^ (7.45-20.99)	4.50^b^ (1.94-10.42)	6.68^b^ (4.21-10.59)	12.97^b^ (3.71-45.29)
	Sumatra	7.37^b^ (4.71-11.54)	7.40^b^ (4.37-12.55)	8.25^b^ (3.55-19.15)	5.52^b^ (3.44-8.85)	13.39^b^ (3.82-46.92)
	Kalimantan	5.54^b^ (3.46-8.86)	4.00^b^ (2.24-7.12)	6.09^b^ (2.60-14.29)	6.50^b^ (3.98-10.61)	1.73 (0.44-6.89)
	Sulawesi	1.73^a^ (1.03-2.93)	0.77 (0.35-1.69)	2.71^a^ (1.12-6.54)	1.36 (0.77-2.40)	2.13 (0.55-8.23)
**Services**						
	Inpatient	Ref	Ref	Ref	Ref	Ref
	Outpatient	3.74^b^ (3.36-4.17)	3.11^b^ (2.71-3.57)	3.22^b^ (2.72-3.82)	2.95^b^ (2.49-3.49)	3.82^b^ (3.27-4.45)
**Hospital ownership**						
	Government	Ref	Ref	Ref	Ref	Ref
	Private	1.30^b^ (1.13-1.50)	0.99 (0.83-1.19)	1.59^b^ (1.28-1.98)	—	—
**Facility type**						
	Clinic	Ref	Ref	Ref	Ref	Ref
	Hospital	0.04^b^ (0.02-0.07)	0.02^b^ (0.01-0.05)	0.06^b^ (0.02-0.15)	5.04 (0.58-43.98)	0.01^b^ (0.00-0.02)
**Facility level**						
	Hospital level A	Ref	Ref	Ref	Ref	Ref
	Hospital level B	0.97 (0.74-1.26)	0.66^a^ (0.47-0.92)	1.32 (0.86-2.04)	0.65^b^ (0.49-0.87)	8.13^b^ (2.12-31.16)
	Hospital level C	0.48^b^ (0.35-0.64)	0.56^b^ (0.39-0.81)	0.38^b^ (0.24-0.61)	0.69^a^ (0.51-0.94)	1.91 (0.49-7.41)
	Hospital level D	0.56^b^ (0.39-0.80)	0.81 (0.50-1.31)	0.39^b^ (0.22-0.67)	0.00^b^ (0.00-0.00)	3.61 (0.92-14.19)
	Others	0.57^b^ (0.41-0.81)	0.52^b^ (0.34-0.80)	0.55^a^ (0.31-0.95)	0.84 (0.58-1.21)	0.58 (0.12-2.82)

^a^*P*<.05.

^b^*P*<.01.

^c^Not applicable.

^d^NT: Nusa Tenggara.

### Disparities in Receiving Hemodialysis Among BPJS Members

Tables 2 and 3 examine the associations between receiving hemodialysis across BPJS membership types for total renal failure claims, by subgroups, such as sex, facility ownership, urbanicity, and region. Compared with individuals in the lowest income group (nationally subsidized members), locally subsidized members and informal worker categories had significantly higher odds of receiving hemodialysis, with aORs of 1.31 (95% CI 1.05-1.63) and 1.56 (95% CI 1.34-1.82), respectively. However, members in the formal worker group had significantly lower odds of receiving hemodialysis (aOR 0.81, 95% CI 0.68-0.98; Table 2).

By sex, the disparity between individuals in the lowest income group (nationally subsidized members) and locally subsidized members was statistically significant among men (aOR 1.72, 95% CI 1.29-2.29) but not among women. In contrast, the disparity between nationally subsidized members and informal workers was significant for both sexes, with a notably larger disparity among women (aORs of 1.27, 95% CI 1.05-1.53, for men and 2.51, 95% CI 1.94-3.23, for women).

By facility ownership, the disparity between the nationally and locally subsidized members was significant in both government and private health facilities, with government facilities showing slightly higher odds of receiving hemodialysis (aORs of 1.76 [1.34-2.31] and 1.48 [1.06-2.06] for government and private facilities, respectively). However, the disparity between nationally subsidized members and informal workers was statistically significant only in private health facilities (aOR 2.59, 95% CI 2.06-3.27) and not in government facilities.

By urbanicity, the disparity between nationally and locally subsidized members was significant in both urban and rural settings, with rural areas showing higher odds of receiving hemodialysis (aORs of 1.69 [1.14-2.50] and 2.40 [1.78-3.23], respectively). The disparity between nationally subsidized members and informal workers was also statistically significant in both urban and rural areas, with aORs of 1.62 [1.21-2.17] and 1.73 [1.41-2.12], respectively. Additionally, the disparity between nationally subsidized members and informal nonworkers was significant only in urban areas (aOR 1.46, 95% CI 1.02-2.09; Table 3).

By region, the disparity between the nationally and locally subsidized members groups was significant in both Java or Bali and non-Java or non-Bali regions, with slightly higher odds observed in the non-Java or non-Bali regions (aORs of 1.46, 95% CI 1.08-1.98, and 1.76, 95% CI 1.31-2.37, respectively). The disparity between nationally subsidized members and informal workers was significant only in Java or Bali (aOR 2.20, 95% CI 1.82-2.67) and not in non-Java or non-Bali regions. Furthermore, the disparity between nationally subsidized members and formal workers was significant exclusively in non-Java or non-Bali regions (aOR 0.61, 95% CI 0.47-0.79).

**Table 2:** Adjusted odds ratios (aORs) of receiving hemodialysis among Badan Penyelenggara Jaminan Sosial (BPJS) members with renal failure, by sex and facility ownership in Indonesia between 2017 and 2022 (N=38,383).

Variable		Total, aOR (95% CI)	Male (n=21,908), aOR (95% CI)	Female (n=16,475), aOR (95% CI)	Government (n=16,828), aOR (95% CI)	Private (n=21,555), aOR (95% CI)
**Membership**						
	Nationally subsidized	Ref	Ref	Ref	Ref	Ref
	Locally subsidized	1.31^a^ (1.05-1.63)	1.72^b^ (1.29-2.29)	1.24 (0.89-1.72)	1.76^b^ (1.34-2.31)	1.48^a^ (1.06-2.06)
	Informal nonworker	1.07 (0.87-1.32)	0.99 (0.72-1.36)	1.34 (0.98-1.85)	0.72 (0.52-1.01)	1.62^b^ (1.22-2.16)
	Informal worker	1.56^b^ (1.34-1.82)	1.27^a^ (1.05-1.53)	2.51^b^ (1.94-3.23)	1.21 (1.00-1.48)	2.59^b^ (2.06-3.27)
	Formal worker	0.81^a^ (0.68-0.98)	0.79 (0.62-1.01)	1.06 (0.78-1.45)	0.81 (0.62-1.04)	1.18 (0.90-1.55)
**Data year**						
	2017	Ref	Ref	Ref	Ref	Ref
	2018	0.80^b^ (0.67-0.94)	0.72^b^ (0.58-0.90)	0.82 (0.64-1.06)	0.91 (0.72-1.16)	0.71^a^ (0.54-0.92)
	2019	0.83^a^ (0.71-0.98)	0.69^b^ (0.56-0.87)	0.89 (0.69-1.16)	0.93 (0.73-1.19)	0.78^a^ (0.60-1.00)
	2020	0.81^a^ (0.67-0.97)	0.82 (0.65-1.04)	0.72^a^ (0.54-0.95)	0.66^b^ (0.48-0.90)	0.83 (0.64-1.08)
	2021	0.76^b^ (0.63-0.91)	0.76^a^ (0.60-0.98)	0.74^a^ (0.55-0.99)	0.87 (0.66-1.14)	0.62^b^ (0.46-0.82)
	2022	0.97 (0.81-1.16)	1.07 (0.85-1.35)	0.92 (0.70-1.22)	0.98 (0.77-1.25)	0.99 (0.74-1.31)
**Sex**						
	Female	Ref	Ref	Ref	Ref	Ref
	Male	1.17^b^ (1.04-1.32)	—	—	1.38^b^ (1.14-1.66)	1.24^b^ (1.07-1.45)
**Age group (years)**						
	18-39	Ref	Ref	Ref	Ref	Ref
	40-49	1.37^b^ (1.16-1.63)	1.69^b^ (1.31-2.17)	1.10 (0.88-1.37)	1.35^a^ (1.04-1.75)	1.26 (0.99-1.59)
	50-59	0.90 (0.76-1.07)	1.45^b^ (1.17-1.80)	0.58^b^ (0.45-0.74)	1.08 (0.86-1.38)	0.54^b^ (0.43-0.68)
	≥60	0.74^b^ (0.63-0.86)	0.97 (0.77-1.21)	0.54^b^ (0.43-0.68)	0.83 (0.65-1.04)	0.62^b^ (0.50-0.77)
**Urbanicity**						
	Rural	Ref	Ref	Ref	Ref	Ref
	Urban	1.07 (0.92-1.24)	1.04 (0.86-1.27)	1.08 (0.85-1.38)	1.42^b^ (1.17-1.72)	0.73^b^ (0.58-0.91)
**Region**						
	Papua, Maluku, NT	Ref	Ref	Ref	Ref	Ref
	Java, Bali	8.30^b^ (5.33-12.94)	12.50^b^ (7.45-20.99)	4.50^b^ (1.94-10.42)	6.68^b^ (4.21-10.59)	12.97^b^ (3.71-45.29)
	Sumatra	7.37^b^ (4.71-11.54)	7.40^b^ (4.37-12.55)	8.25^b^ (3.55-19.15)	5.52^b^ (3.44-8.85)	13.39^b^ (3.82-46.92)
	Kalimantan	5.54^b^ (3.46-8.86)	4.00^b^ (2.24-7.12)	6.09^b^ (2.60-14.29)	6.50^b^ (3.98-10.61)	1.73 (0.44-6.89)
	Sulawesi	1.73^a^ (1.03-2.93)	0.77 (0.35-1.69)	2.71^a^ (1.12-6.54)	1.36 (0.77-2.40)	2.13 (0.55-8.23)
**Services**						
	Inpatient	Ref	Ref	Ref	Ref	Ref
	Outpatient	3.74^b^ (3.36-4.17)	3.11^b^ (2.71-3.57)	3.22^b^ (2.72-3.82)	2.95^b^ (2.49-3.49)	3.82^b^ (3.27-4.45)
**Hospital ownership**						
	Government	Ref	Ref	Ref	Ref	Ref
	Private	1.30^b^ (1.13-1.50)	0.99 (0.83-1.19)	1.59^b^ (1.28-1.98)	—	—
**Facility type**						
	Clinic	Ref	Ref	Ref	Ref	Ref
	Hospital	0.04^b^ (0.02-0.07)	0.02^b^ (0.01-0.05)	0.06^b^ (0.02-0.15)	5.04 (0.58-43.98)	0.01^b^ (0.00-0.02)
**Facility level**						
	Hospital level A	Ref	Ref	Ref	Ref	Ref
	Hospital level B	0.97 (0.74-1.26)	0.66^a^ (0.47-0.92)	1.32 (0.86-2.04)	0.65^b^ (0.49-0.87)	8.13^b^ (2.12-31.16)
	Hospital level C	0.48^b^ (0.35-0.64)	0.56^b^ (0.39-0.81)	0.38^b^ (0.24-0.61)	0.69^a^ (0.51-0.94)	1.91 (0.49-7.41)
	Hospital level D	0.56^b^ (0.39-0.80)	0.81 (0.50-1.31)	0.39^b^ (0.22-0.67)	0.00^b^ (0.00-0.00)	3.61 (0.92-14.19)
	Others	0.57^b^ (0.41-0.81)	0.52^b^ (0.34-0.80)	0.55^a^ (0.31-0.95)	0.84 (0.58-1.21)	0.58 (0.12-2.82)

^a^*P*<.05.

^b^*P*<.01.

^c^NT: Nusa Tenggara.

^d^Not applicable.

**Table 3:** Adjusted odds ratios (aORs) of receiving hemodialysis among Badan Penyelenggara Jaminan Sosial (BPJS) members with renal failure, by urbanicity and region in Indonesia between 2017 and 2022.

Variable		Urban, aOR (95% CI)	Rural, aOR (95% CI)	Java, aOR (95% CI)	Non-Java, aOR (95% CI)
**Membership**					
	Nationally subsidized	Reference	Reference	Reference	Reference
	Locally subsidized	1.69^a^ (1.14-2.50)	2.40^a^ (1.78-3.23)	1.46^b^ (1.08-1.98)	1.76^a^ (1.31-2.37)
	Informal nonworker	1.46^b^ (1.02-2.09)	0.78 (0.56-1.09)	1.10 (0.85-1.43)	1.43 (1.00-2.07)
	Informal worker	1.62^a^ (1.21-2.17)	1.73^a^ (1.41-2.12)	2.20^a^ (1.82-2.67)	1.02 (0.81-1.28)
	Formal worker	0.96 (0.69-1.33)	0.87 (0.67-1.13)	0.86 (0.68-1.09)	0.61^a^ (0.47-0.79)
**Data year**					
	2017	Reference	Reference	Reference	Reference
	2018	0.71^a^ (0.55-0.91)	0.94 (0.74-1.19)	0.82^b^ (0.67-0.99)	0.76 (0.58-1.01)
	2019	0.73^b^ (0.57-0.93)	1.02 (0.79-1.30)	0.92 (0.75-1.12)	0.77 (0.59-1.02)
	2020	0.52^a^ (0.40-0.68)	1.10 (0.82-1.48)	0.81 (0.65-1.01)	0.94 (0.71-1.25)
	2021	0.51^a^ (0.39-0.66)	1.22 (0.93-1.61)	0.83 (0.65-1.04)	0.93 (0.70-1.24)
	2022	0.74^b^ (0.57-0.97)	1.32^b^ (1.03-1.68)	1.08 (0.87-1.35)	1.04 (0.78-1.38)
**Sex**					
	Female	Reference	Reference	Reference	Reference
	Male	0.85 (0.72-1.00)	1.77^a^ (1.46-2.14)	1.75^a^ (1.50-2.03)	0.66^a^ (0.56-0.78)
**Age group**					
	18-39 years	Reference	Reference	Reference	Reference
	40-49 years	1.08 (0.85-1.37)	1.60^a^ (1.25-2.05)	0.86 (0.70-1.07)	2.07^a^ (1.58-2.72)
	50-59 years	0.66^a^ (0.52-0.85)	1.17 (0.93-1.49)	0.71^a^ (0.57-0.87)	0.86 (0.66-1.11)
	60+ years	0.68^a^ (0.54-0.85)	0.82 (0.65-1.04)	0.51^a^ (0.42-0.63)	0.78 (0.59-1.03)
**Urbanicity**					
	Rural	Reference	Reference	Reference	Reference
	Urban	—^c^	—	1.19 (0.97-1.45)	0.91 (0.73-1.14)
**Region**					
	Papua, Maluku, NT^d^	Reference	Reference	Reference	Reference
	Java, Bali	8.77^a^ (5.50-13.98)	8.46^a^ (3.20-22.33)	—	—
	Sumatra	9.02^a^ (5.58-14.57)	6.95^a^ (2.65-18.25)	—	—
	Kalimantan	7.56^a^ (4.53-12.60)	2.96^b^ (1.07-8.18)	—	—
	Sulawesi	3.67^a^ (2.13-6.34)	0.21^b^ (0.06-0.75)	—	—
**Services**					
	Inpatient	Reference	Reference	Reference	Reference
	Outpatient	2.75^a^ (2.35-3.22)	3.05^a^ (2.54-3.67)	3.11^a^ (2.71-3.58)	3.87^a^ (3.26-4.59)
**Hospital ownership**					
	Government	Reference	Reference	Reference	Reference
	Private	1.22 (0.99-1.49)	0.97 (0.78-1.19)	1.32^a^ (1.10-1.57)	1.34^a^ (1.10-1.64)
**Facility type**					
	Clinic	Reference	Reference	Reference	Reference
	Hospital	0.10^a^ (0.04-0.24)	0.02^a^ (0.01-0.04)	0.05^a^ (0.03-0.10)	0.01^a^ (0.00-0.02)
**Facility level**					
	Hospital level A	Reference	Reference	Reference	Reference
	Hospital level B	1.43^b^ (1.07-1.92)	2.05 (0.44-9.56)	2.00^a^ (1.36-2.94)	0.35^a^ (0.25-0.49)
	Hospital level C	0.26^a^ (0.19-0.36)	3.40 (0.72-16.00)	0.85 (0.56-1.31)	0.24^a^ (0.17-0.35)
	Hospital level D	0.07^a^ (0.02-0.18)	4.30 (0.91-20.44)	1.57 (0.95-2.60)	0.01^a^ (0.01-0.03)
	Others	0.51^a^ (0.36-0.74)	3.13 (0.63-15.48)	1.29 (0.82-2.02)	0.19^a^ (0.11-0.33)

^a^*P*<.01.

^a^*P*<.05.

^c^Not applicable.

^d^NT: Nusa Tenggara.

### Disparities in Access to Hemodialysis Among BPJS Members Across Covariates

Within Indonesia’s national health insurance system, men demonstrated significantly higher odds of receiving hemodialysis compared with women (aOR 1.17, 95% CI 1.04-1.32). However, age-related disparities were more nuanced. Compared with the youngest age group (18-30 years), individuals aged 40 to 49 years were significantly more likely to receive hemodialysis (aOR 1.37, 95% CI 1.16-1.63), while older adults aged ≥60 years were less likely (aOR 0.74, 95% CI 0.63-0.86; Table 2).

Regarding urbanicity, although patients in urban areas had slightly higher rates of receiving hemodialysis, the difference was not statistically significant in the multivariate analysis (Table 2). Regional disparities, however, were notable. Compared with members residing in the least advantaged region (Papua, Maluku, and Nusa Tenggara), those living in other regions had significantly greater odds of accessing hemodialysis, with particularly high odds observed in Java or Bali, Sumatra, and Kalimantan (aORs of 8.30, 95% CI 5.33-12.94, 7.37, 95% CI 4.71-11.54, and 5.54, 95% CI 3.46-8.86, respectively).

Facility-level characteristics also played a significant role. Patients using outpatient services were more likely to receive hemodialysis compared with those in inpatient services (aOR 3.74, 95% CI 3.36-4.17). Furthermore, patients treated at private facilities had higher odds of accessing hemodialysis than those at government-run facilities (aOR 1.30, 95% CI 1.13-1.50). When examining at the facility level, patients in hospitals classified below level A had lower odds of receiving hemodialysis, with significant disparities evident for those treated at level C and level D hospitals, as well as in other types of facilities, such as clinics and specialized hemodialysis centers.

## Discussion

### Overview

Our study highlights significant socioeconomic, demographic, and facility-level disparities in hemodialysis claim patterns, which—within the context of near-universal BPJS coverage—can be considered a reasonable proxy for disparities in access to renal care among members diagnosed with renal failure in Indonesia. Using national health insurance claims data from 2017 to 2022, we identified inequities across membership types, sex, age, urbanicity, region, and facility characteristics. These findings underscore the need for targeted interventions to address barriers to equitable health care access, particularly in lower socioeconomic groups and underserved regions.

### Socioeconomic Disparities

The disparities observed in hemodialysis access across BPJS membership types reflect broader inequities within Indonesia’s health care system. Informal workers had higher odds of receiving hemodialysis compared with those in the lowest income group (nationally subsidized members). This suggests that while BPJS coverage reduces financial barriers for subsidized members, other systemic obstacles—such as accessibility and awareness—may disproportionately affect the lowest income groups. Prior studies have similarly reported that socioeconomically disadvantaged populations often face multiple barriers to accessing specialized health care services, even under universal health coverage schemes [7,10,17,18].

In contrast, formal workers had lower odds of receiving hemodialysis, which may reflect competing demands of employment that limit their ability to access treatment. This aligns with research indicating that time and resource constraints among employed populations can adversely impact health care use [25]. Policymakers should consider tailored strategies, such as workplace health initiatives, to mitigate these challenges.

### Demographic and Regional Disparities

Demographic analysis revealed notable sex-based disparities, with men being more likely to receive hemodialysis than women. This finding echoes global trends where men often exhibit higher health care use for certain conditions, potentially because of physiological differences in disease progression or social factors influencing health-seeking behavior [26]. Age-based disparities were also evident, with younger patients more likely to access hemodialysis compared with older adults. This aligns with findings from other LMICs where older populations face higher treatment discontinuation rates due to financial, logistical, or health-related constraints [27].

Regional disparities were particularly stark, with patients in Java or Bali having significantly higher odds of receiving hemodialysis compared with those in Papua, Maluku, and Nusa Tenggara. Java or Bali’s well-developed health care infrastructure likely contributes to this advantage, while the limited availability of specialized facilities in less advantaged regions hinders access. Previous studies have documented similar regional and urban-rural divides in health care access, emphasizing the need for infrastructure investments to bridge these gaps [20,28].

### Facility-Level Factors

Facility-level factors played a crucial role in determining access to hemodialysis. Outpatient services were significantly associated with higher odds of receiving hemodialysis compared with inpatient settings. This may reflect the chronic nature of renal failure, where frequent outpatient visits for dialysis are standard. However, reliance on outpatient care may disadvantage patients who lack consistent transportation or proximity to facilities, as observed in other studies on chronic disease management [29]. Facility ownership and level also influenced access disparities. Patients treated at private facilities were more likely to receive hemodialysis than those in government-run hospitals. This finding highlights potential differences in service capacity or quality between private and public providers, a concern frequently raised in studies on dual-sector health care systems [6,30]. The consistently lower odds of hemodialysis claims observed in hospitals below level A likely reflect the referral system in Indonesia, where the most severe and complex renal failure cases are concentrated at national referral (level A) hospitals; this pattern may therefore indicate appropriate care allocation rather than inequality, while disparities across regions and facility types may still contribute to variation in access [11,23].

For policy, to address the observed disparities in hemodialysis access, policy makers should consider targeted interventions. Outreach programs focused on subsidized members, particularly in rural and underserved regions, can help overcome geographic and financial barriers through expanded transportation assistance, public education campaigns, and community-based dialysis centers. Strengthening government health facilities with investments in equipment, staffing, and training is essential to improving service quality and equity, while public-private partnerships could enhance service delivery and affordability [6]. In Indonesia, dialysis services remain overwhelmingly hospital-based, with the Indonesia Renal Registry reporting that 98% of patients receive hemodialysis while only 2% use continuous ambulatory peritoneal dialysis, despite continuous ambulatory peritoneal dialysis being cost-saving and fully covered by BPJS [31]. This underuse indicates that alternative modalities and service adaptations—such as home-based or mobile dialysis and flexible scheduling—are insufficiently implemented. Our analysis also showed that formal sector workers had lower odds of hemodialysis claims compared with other groups, which may reflect work-related barriers. Addressing these challenges could involve piloting flexible work policies and mobile dialysis units, supported by evidence that flexible scheduling helps patients maintain employment and that mobile services can reduce transport-related barriers [31,32]. Finally, raising awareness about renal failure management among patients and health care providers can improve timely recognition and management of the condition [13,14].

This study has several limitations. First, this study focuses on claims involving hemodialysis, which accounts for over 90% of dialysis-related procedures in the dataset. While peritoneal dialysis and transplantation are technically available in Indonesia, their uptake is extremely limited and inconsistently captured in claims data, which may result in the exclusion of a small subset of patients receiving alternative modalities [5,13]. Second, claims data may not capture all cases of renal failure, particularly among uninsured or underdiagnosed populations. Third, while aORs provide insights into associations, causal relationships cannot be inferred. Future research should explore longitudinal data to better understand the dynamics of hemodialysis access over time.

While delays in care-seeking and treatment initiation are plausible concerns in Indonesia’s health system, our claim-level data do not allow us to measure patient-level outcomes, such as time to first dialysis, missed sessions, or referral delays. As such, these issues cannot be empirically confirmed in this study and should be addressed in future research using longitudinal or patient-level data.

### Conclusions

This study provides critical insights into socioeconomic, demographic, and facility-level disparities in hemodialysis claim patterns among BPJS members in Indonesia. Hemodialysis was provided in 75.6% (29,017/38,383) of renal failure claims. Nationally subsidized members had significantly lower odds of receiving hemodialysis compared with informal workers and locally subsidized members, with aORs of 1.56 and 1.31, respectively. Disparities were more pronounced in rural areas, where locally subsidized members had higher odds (aOR 2.40) than their nationally subsidized counterparts. Patients in private facilities (aOR 1.30) and urban areas had better access, while regional disparities favored developed regions like Java or Bali.

While these findings highlight meaningful inequities, it is important to note that our dataset is claim-based and does not permit direct assessment of patient-level outcomes, such as time to first dialysis, missed sessions, or referral delays. Future studies incorporating longitudinal patient trajectories are needed to examine these dynamics more precisely. Nevertheless, the disparities identified here underscore the need for coordinated policy action. Strengthening health care infrastructure, expanding community-based and mobile dialysis services, improving workplace flexibility for formal sector workers, and reducing barriers for subsidized and rural populations are critical steps. By implementing targeted interventions, Indonesia can make significant progress toward universal and equitable access to essential renal care services.

## Appendix

Multimedia Appendix 1 The flowchart outlining the sample selection process.

Multimedia Appendix 2 Total number of claims and unique patients by year.

## Abbreviations

aOR: adjusted odds ratio
BPJS: Badan Penyelenggara Jaminan Sosial
CKD: chronic kidney disease
ESRD: end-stage renal disease
ICD: International Classification of Diseases
LMIC: low- and middle-income country

## Data Availability

The dataset used in this study is available from the authors upon reasonable request.

## References

[1] Kovesdy CP (2022). Epidemiology of chronic kidney disease: an update 2022. Kidney Int Suppl (2011).

[2] GBD 2021 Causes of Death Collaborators (2024). Global burden of 288 causes of death and life expectancy decomposition in 204 countries and territories and 811 subnational locations, 1990-2021: a systematic analysis for the Global Burden of Disease Study 2021. Lancet.

[3] GBD Chronic Kidney Disease Collaboration (2020). Global, regional, and national burden of chronic kidney disease, 1990-2017: a systematic analysis for the Global Burden of Disease Study 2017. Lancet.

[4] VizHub - GBD results. Institute for Health Metrics and Evaluation.

[5] Violetta L, Kusumaningrum VF, Jonny (2022). Peritoneal dialysis in Indonesia: current status, challenges and prospects. Perit Dial Int.

[6] Hyodo T, Hirawa N, Kuragano T, Takemoto Y, Urabe S, Adiya S, Damdinsuren K, Munaa B, Yondontsamts A, Jamba A, Dolgorsuren M, Maamkhuu O, Dorj C, Widiana IG (2025). The present status of dialysis patients in Asian countries as of 2022 from a medical economic point of view. Ren Replace Ther.

[7] Nugroho ST, Ahsan A, Kusuma D, Adani N, Irawaty DK, Amalia N, Hati SR (2023). Income disparity and healthcare utilization: lessons from Indonesia’s national health insurance claim data. Asian Pac J Cancer Prev.

[8] Darmawan ES, Permanasari VY, Nisrina LV, Kusuma D, Hasibuan SR, Widyasanti N (2024). Behind the hospital ward: in-hospital mortality of type 2 diabetes mellitus patients in Indonesia (analysis of national health insurance claim sample data). Int J Environ Res Public Health.

[9] Untari PH (2024). BPJS Kesehatan bayar klaim paling besar untuk 8 penyakit sepanjang 2023, capai Rp34,7 triliun. Bisnis.

[10] Bachtiar A, Candi C, Hasibuan SR, Widyasanti N, Kusuma D (2024). Navigating the aftermath: risk factors of recurrence following coronary bypass surgery in Indonesia. Narra J.

[11] Darmawan ES, Hasibuan SR, Permanasari VY, Kusuma D (2025). Disparities in health services and outcomes by National Health Insurance membership type for ischemic heart disease and stroke in Indonesia: analysis of claims, 2017-2022. Glob Health Res Policy.

[12] Mahendradhata Y, Trisnantoro L, Listyadewi S, Soewondo P, Marthias T, Harimurti P, Prawira J (2017). The Republic of Indonesia health system review. World Health Organization.

[13] Bello AK, Okpechi IG, Levin A, Ye F, Damster S, Arruebo S, Donner JA, Caskey FJ, Cho Y, Davids MR, Davison SN, Htay H, Jha V, Lalji R, Malik C, Nangaku M, See E, Sozio SM, Tonelli M, Wainstein M, Yeung EK, Johnson DW, ISN-GKHA Group (2024). An update on the global disparities in kidney disease burden and care across world countries and regions. Lancet Glob Health.

[14] Francis A, Harhay MN, Ong AC, Tummalapalli SL, Ortiz A, Fogo AB, Fliser D, Roy-Chaudhury P, Fontana M, Nangaku M, Wanner C, Malik C, Hradsky A, Adu D, Bavanandan S, Cusumano A, Sola L, Ulasi I, Jha V, American Society of Nephrology, European Renal Association, International Society of Nephrology (2024). Chronic kidney disease and the global public health agenda: an international consensus. Nat Rev Nephrol.

[15] Andriani H, Rahmawati ND, Ahsan A, Kusuma D (2023). Secondhand smoke exposure inside the house and low birth weight in Indonesia: evidence from a demographic and health survey. Popul Med.

[16] Oktamianti P, Kusuma D, Amir V, Tjandrarini DH, Paramita A (2023). Does the disparity patterning differ between diagnosed and undiagnosed hypertension among adults? Evidence from Indonesia. Healthcare (Basel).

[17] Park HC, Kwon YE, Choi HY, Oh HJ, Chang TI, Kang EW, Park KS, Yang KH, Won EM, Shin JH, Ryu DR, Lee YK (2020). Health insurance status is related to risk of mortality and hospitalization in Korean maintenance hemodialysis patients: a longitudinal cohort study. Am J Nephrol.

[18] Ke C, Kim SJ, Shah BR, Bierman AS, Lipscombe LL, Feig DS, Booth GL (2019). Impact of socioeconomic status on incidence of end-stage renal disease and mortality after dialysis in adults with diabetes. Can J Diabetes.

[19] Shankar M, Sankarasubaiyan S, Kasiviswanathan S, Shah KD, Luyckx V (2024). Gender disparity in hemodialysis practices and mortality: a nationwide cross-sectional observational study. Indian J Nephrol.

[20] Sulistiadi W, Kusuma D, Amir V, Tjandrarini DH, Nurjana MA (2023). Growing up unequal: disparities of childhood overweight and obesity in Indonesia’s 514 districts. Healthcare (Basel).

[21] Darmawan ES, Kusuma D, Permanasari VY, Amir V, Tjandrarini DH, Dharmayanti I (2023). Beyond the plate: uncovering inequalities in fruit and vegetable intake across Indonesian districts. Nutrients.

[22] Setiawan E, Nurjannah N, Komaryani K, Nugraha RR, Thabrany H, Purwaningrum F, Sarnianto P (2022). Utilization patterns of healthcare facility and estimated expenditure of PLHIV care under the Indonesian National Health Insurance Scheme in 2018. BMC Health Serv Res.

[23] Darmawan ES, Hasibuan SR, Permanasari VY, Kusuma D (2025). Disparities in cancer care and outcomes by insurance membership type in Indonesia: a retrospective cross-sectional analysis of national health insurance claims, 2017-2022. BMJ Open.

[24] (2016). ICD-10 version: 2016. World Health Organization.

[25] Tsutsui H, Nomura K, Ishiguro A, Tsuruta Y, Kato S, Yasuda Y, Uchida S, Oshida Y (2017). Factors associated with employment in patients undergoing hemodialysis: a mixed methods study. Ren Replace Ther.

[26] Hecking M, Bieber BA, Ethier J, Kautzky-Willer A, Sunder-Plassmann G, Säemann MD, Ramirez SP, Gillespie BW, Pisoni RL, Robinson BM, Port FK (2014). Sex-specific differences in hemodialysis prevalence and practices and the male-to-female mortality rate: the Dialysis Outcomes and Practice Patterns Study (DOPPS). PLoS Med.

[27] Tonelli M, Nkunu V, Varghese C, Abu-Alfa AK, Alrukhaimi MN, Fox L, Gill J, Harris DC, Hou FF, O'Connell PJ, Rashid HU, Niang A, Ossareh S, Tesar V, Zakharova E, Yang CW (2020). Framework for establishing integrated kidney care programs in low- and middle-income countries. Kidney Int Suppl (2011).

[28] Megatsari H, Kusuma D, Ernawaty E, Putri NK (2022). Geographic and socioeconomic inequalities in delays in COVID-19 vaccinations: a cross-sectional study in Indonesia. Vaccines (Basel).

[29] Starbird LE, DiMaina C, Sun CA, Han HR (2019). A systematic review of interventions to minimize transportation barriers among people with chronic diseases. J Community Health.

[30] Septiono W (2023). Equity challenges in Indonesian health care. Lancet Glob Health.

[31] Putri S, Nugraha RR, Pujiyanti E, Thabrany H, Hasnur H, Istanti ND, Evasari D (2022). Supporting dialysis policy for end stage renal disease (ESRD) in Indonesia: an updated cost-effectiveness model. BMC Res Notes.

[32] Liu MW, Syukri M, Abdullah A, Chien LY (2021). Missing in-center hemodialysis sessions among patients with end stage renal disease in Banda Aceh, Indonesia. Int J Environ Res Public Health.

