# Supplementary material for: Differences in Hemodialysis Claim Patterns Across Membership Types Among Patients With Renal Failure Based on National Health Insurance Data From 2017 to 2022: Cross-Sectional Analysis
Source: JMIR Public Health Surveill. 2025 Nov 3;11:e73731. doi: 10.2196/73731 (PMC12624297; doi:10.2196/73731)
Supplement: Multimedia Appendix 1 [file publichealth_v11i1e73731_app1.docx]

**Appendix 1. The flowchart outlining the sample selection process**

Sampel klaim

| 6,897,148 | |  | Total claims 2017-2022 |
| --- | --- | --- | --- |
|  |  |  | 6,858,361 claims not ICD-10 N17 - N19 removed |
|  |  |  |  |
| 38,787 | |  |  |
|  |  |  | 404 claims of samples < 18 years old removed |
|  |  |  |  |
| 38,383 | |  | Final sample |

**Appendix 2. Total Number of Claims and Unique Patients by Year in the Sample Dataset, Indonesia (2017–2022)**

| **Year** | **Total Claims** | **Unique Patients** |
| --- | --- | --- |
| 2017 | 5,262 | 974 |
| 2018 | 6,423 | 1,246 |
| 2019 | 6,683 | 1,411 |
| 2020 | 6,366 | 1,375 |
| 2021 | 6,390 | 1,321 |
| 2022 | 7,259 | 1,589 |
| Total | 38,383 | 7,916 |

Note: Only a small proportion of patients had repeated claims across years. For example, just 30.7% of patients identified in 2017 reappeared in 2018, and this proportion declined further in subsequent years. This limited overlap supports the use of a cross-sectional, claim-level approach rather than a panel analysis. See Appendix 2.

**Appendix 2: Unadjusted regressions among BPJS members with all cancer**

|  | Biopsy | | Transfusion | | Severity | | Mortality | | Length of stay | | Claim cost (Ln) | |
| --- | --- | --- | --- | --- | --- | --- | --- | --- | --- | --- | --- | --- |
| Variables | AOR | (SE) | AOR | (SE) | AOR | (SE) | AOR | (SE) | Coef | (SE) | Coef | (SE) |
|  |  |  |  |  |  |  |  |  |  |  |  |  |
| Membership |  |  |  |  |  |  |  |  |  |  |  |  |
| PBI APBN | Ref |  | Ref |  | Ref |  | Ref |  | Ref |  | Ref |  |
| PBI APBD | 0.68* | (0.12) | 1.20 | (0.17) | 1.11 | (0.13) | 0.98 | (0.26) | 0.36 | (0.22) | 0.07* | (0.03) |
| Informal non-worker (BP) | 1.20 | (0.28) | 2.07** | (0.28) | 2.03** | (0.22) | 1.92** | (0.38) | 1.26** | (0.29) | 0.44** | (0.03) |
| Informal worker (PBPU) | 0.89 | (0.11) | 1.48** | (0.13) | 1.26** | (0.09) | 1.06 | (0.17) | 0.25* | (0.11) | 0.21** | (0.02) |
| Formal worker (PPU) | 0.76* | (0.10) | 0.90 | (0.09) | 0.81** | (0.06) | 0.74 | (0.13) | -0.29** | (0.11) | 0.32** | (0.02) |
|  |  |  |  |  |  |  |  |  |  |  |  |  |
| Constant | 0.09** | (0.01) | 0.15** | (0.01) | 0.30** | (0.02) | 0.05** | (0.01) | 3.66** | (0.09) | 15.42** | (0.01) |
| Observations | 34,904 |  | 34,904 |  | 34,904 |  | 34,904 |  | 34,904 |  | 34,904 |  |

Note: AOR=Adjusted Odds Ratio, Coef=Coefficient, SE=Standard errors, Ref=Reference, PBI= Subsidized members, APBN=National government budget, APBD=Local government budget, Ln=Natural log. Transfusion included transfusion of packed cells and transfusion of platelets; Pooled logit regressions for AORs and OLS regressions for Coefs in Stata 15. Robust SE in parentheses. ** p<0.01, * p<0.05

**Appendix 3: Correlates of health services among BPJS members with all cancer, Indonesia 2017-2022**

|  | Biopsy | | Transfusion | | Severity | | Mortality | | LOS | | Claim cost (Ln) | |
| --- | --- | --- | --- | --- | --- | --- | --- | --- | --- | --- | --- | --- |
| Variables | AOR | (SE) | AOR | (SE) | AOR | (SE) | AOR | (SE) | Coef | (SE) | Coef | (SE) |
|  |  |  |  |  |  |  |  |  |  |  |  |  |
| Membership |  |  |  |  |  |  |  |  |  |  |  |  |
| PBI APBN | Ref |  | Ref |  | Ref |  | Ref |  | Ref |  | Ref |  |
| PBI APBD | 0.62** | (0.11) | 1.01 | (0.15) | 0.92 | (0.12) | 0.74 | (0.19) | 0.02 | (0.18) | -0.02 | (0.02) |
| Informal non-worker (BP) | 0.93 | (0.21) | 1.43* | (0.22) | 1.26 | (0.15) | 1.17 | (0.26) | 0.30 | (0.24) | 0.29** | (0.03) |
| Informal worker (PBPU) | 0.79 | (0.10) | 1.26* | (0.12) | 1.06 | (0.08) | 0.82 | (0.13) | -0.07 | (0.09) | 0.10** | (0.01) |
| Formal worker (PPU) | 0.77* | (0.10) | 0.88 | (0.09) | 0.78** | (0.06) | 0.67* | (0.12) | -0.27** | (0.10) | 0.26** | (0.01) |
| Data year |  |  |  |  |  |  |  |  |  |  |  |  |
| 2017 | Ref |  | Ref |  | Ref |  | Ref |  | Ref |  | Ref |  |
| 2018 | 0.95 | (0.15) | 1.24 | (0.14) | 1.16 | (0.11) | 1.19 | (0.21) | 0.01 | (0.12) | -0.01 | (0.01) |
| 2019 | 1.24 | (0.19) | 1.18 | (0.13) | 1.08 | (0.10) | 1.06 | (0.19) | -0.24 | (0.13) | 0.00 | (0.02) |
| 2020 | 1.17 | (0.20) | 1.56** | (0.19) | 1.41** | (0.14) | 1.61* | (0.33) | -0.15 | (0.15) | 0.05** | (0.02) |
| 2021 | 1.75** | (0.27) | 1.59** | (0.18) | 1.58** | (0.15) | 1.59* | (0.30) | 0.01 | (0.14) | 0.06** | (0.02) |
| 2022 | 1.37* | (0.21) | 1.22 | (0.14) | 1.06 | (0.10) | 1.02 | (0.19) | -0.36** | (0.12) | 0.02 | (0.02) |
| Sex |  |  |  |  |  |  |  |  |  |  |  |  |
| Female | Ref |  | Ref |  | Ref |  | Ref |  | Ref |  | Ref |  |
| Male | 0.67** | (0.07) | 0.76** | (0.06) | 0.93 | (0.06) | 1.43** | (0.18) | 0.33** | (0.09) | -0.09** | (0.01) |
| Age group |  |  |  |  |  |  |  |  |  |  |  |  |
| 16-29 years | Ref |  | Ref |  | Ref |  | Ref |  | Ref |  | Ref |  |
| 30-39 years | 0.69* | (0.10) | 1.73** | (0.24) | 2.13** | (0.24) | 1.21 | (0.34) | 0.32** | (0.10) | 0.03 | (0.02) |
| 40-49 years | 0.88 | (0.12) | 3.27** | (0.40) | 3.56** | (0.35) | 2.01** | (0.50) | 0.85** | (0.10) | 0.05** | (0.01) |
| 50-59 years | 0.97 | (0.15) | 3.40** | (0.46) | 3.90** | (0.41) | 1.84* | (0.47) | 0.92** | (0.12) | 0.03 | (0.02) |
| 60+ years | 1.03 | (0.15) | 3.02** | (0.43) | 4.02** | (0.46) | 2.09** | (0.54) | 1.06** | (0.13) | 0.02 | (0.02) |
| Urbanicity |  |  |  |  |  |  |  |  |  |  |  |  |
| Rural | Ref |  | Ref |  | Ref |  | Ref |  | Ref |  | Ref |  |
| Urban | 1.07 | (0.11) | 1.19* | (0.10) | 1.13 | (0.07) | 1.25 | (0.19) | 0.24** | (0.08) | 0.10** | (0.01) |
| Region |  |  |  |  |  |  |  |  |  |  |  |  |
| Papua, Maluku, NT | Ref |  | Ref |  | Ref |  | Ref |  | Ref |  | Ref |  |
| Java, Bali | 1.28 | (0.31) | 0.90 | (0.14) | 0.68** | (0.08) | 0.63* | (0.15) | -0.78** | (0.15) | 0.03 | (0.02) |
| Sumatra | 0.81 | (0.21) | 0.80 | (0.13) | 0.56** | (0.07) | 0.55* | (0.13) | -0.27 | (0.16) | -0.04* | (0.02) |
| Kalimantan | 1.10 | (0.30) | 0.79 | (0.13) | 0.67** | (0.10) | 0.70 | (0.20) | -0.74** | (0.17) | -0.03 | (0.02) |
| Sulawesi | 0.77 | (0.20) | 0.56** | (0.11) | 0.61** | (0.08) | 0.57 | (0.17) | -0.01 | (0.16) | -0.03 | (0.02) |
| Hospital ownership |  |  |  |  |  |  |  |  |  |  |  |  |
| Private | Ref |  | Ref |  | Ref |  | Ref |  | Ref |  | Ref |  |
| Government | 1.25* | (0.14) | 1.40** | (0.11) | 1.52** | (0.10) | 2.01** | (0.32) | 0.91** | (0.08) | -0.01 | (0.01) |
| Hospital level |  |  |  |  |  |  |  |  |  |  |  |  |
| Level A | Ref |  | Ref |  | Ref |  | Ref |  | Ref |  | Ref |  |
| Level B | 0.64** | (0.08) | 0.50** | (0.05) | 0.37** | (0.03) | 0.42** | (0.06) | -2.66** | (0.19) | -0.56** | (0.02) |
| Level C | 0.42** | (0.06) | 0.21** | (0.02) | 0.17** | (0.02) | 0.14** | (0.03) | -4.20** | (0.18) | -0.91** | (0.02) |
| Level D | 0.28** | (0.06) | 0.14** | (0.03) | 0.12** | (0.02) | 0.09** | (0.03) | -4.46** | (0.20) | -1.04** | (0.03) |
| Specialties | 0.54* | (0.13) | 0.77 | (0.13) | 0.83 | (0.12) | 1.08 | (0.31) | -1.80** | (0.46) | -0.18** | (0.06) |
|  |  |  |  |  |  |  |  |  |  |  |  |  |
| Constant | 0.13** | (0.05) | 0.12** | (0.03) | 0.35** | (0.08) | 0.05** | (0.02) | 6.27** | (0.30) | 16.10** | (0.04) |
| Observations | 34,904 |  | 34,904 |  | 34,904 |  | 34,904 |  | 34,904 |  | 34,904 |  |

Note: AOR=Adjusted Odds Ratio, SE=Standard errors, Ref=Reference, NT=Nusa Tenggara, PBI= Subsidized members, APBN=National government budget, APBD=Local government budget, Ln=Natural log. Transfusion included transfusion of packed cells and transfusion of platelets; Pooled logit regressions in Stata 15. Robust SE in parentheses. ** p<0.01, * p<0.05

**Appendix 4: Correlates of health services and outcomes among BPJS members with malignant cancer, Indonesia 2017-2022**

|  | Biopsy | | Transfusion | | Severity | | Mortality | | LOS | | Claim cost (Ln) | |
| --- | --- | --- | --- | --- | --- | --- | --- | --- | --- | --- | --- | --- |
| Variables | AOR | (SE) | AOR | (SE) | AOR | (SE) | AOR | (SE) | Coef | (SE) | Coef | (SE) |
|  |  |  |  |  |  |  |  |  |  |  |  |  |
| Membership |  |  |  |  |  |  |  |  |  |  |  |  |
| PBI APBN | Ref |  | Ref |  | Ref |  | Ref |  | Ref |  | Ref |  |
| PBI APBD | 0.70 | (0.20) | 1.32 | (0.28) | 1.04 | (0.21) | 0.89 | (0.29) | 0.09 | (0.43) | -0.05 | (0.06) |
| Informal non-worker (BP) | 0.96 | (0.31) | 1.69** | (0.33) | 1.69** | (0.30) | 1.26 | (0.35) | 0.66 | (0.50) | 0.28** | (0.04) |
| Informal worker (PBPU) | 0.80 | (0.13) | 1.32* | (0.16) | 1.18 | (0.13) | 0.82 | (0.17) | -0.19 | (0.23) | 0.09** | (0.03) |
| Formal worker (PPU) | 0.76 | (0.16) | 1.15 | (0.17) | 1.11 | (0.15) | 0.76 | (0.19) | 0.08 | (0.28) | 0.30** | (0.04) |
| Data year |  |  |  |  |  |  |  |  |  |  |  |  |
| 2017 | Ref |  | Ref |  | Ref |  | Ref |  | Ref |  | Ref |  |
| 2018 | 0.98 | (0.26) | 1.32 | (0.23) | 1.14 | (0.17) | 1.13 | (0.27) | -0.45 | (0.36) | -0.01 | (0.03) |
| 2019 | 1.17 | (0.31) | 1.04 | (0.16) | 0.90 | (0.12) | 0.91 | (0.21) | -0.92* | (0.37) | -0.00 | (0.04) |
| 2020 | 0.98 | (0.28) | 1.78** | (0.29) | 1.37* | (0.20) | 0.97 | (0.23) | -1.64** | (0.36) | 0.08 | (0.04) |
| 2021 | 1.33 | (0.35) | 1.38* | (0.22) | 1.38* | (0.20) | 1.27 | (0.30) | -0.65 | (0.38) | 0.10** | (0.04) |
| 2022 | 1.27 | (0.32) | 1.10 | (0.16) | 0.89 | (0.12) | 1.02 | (0.23) | -1.20** | (0.36) | 0.03 | (0.03) |
| Sex |  |  |  |  |  |  |  |  |  |  |  |  |
| Female | Ref |  | Ref |  | Ref |  | Ref |  | Ref |  | Ref |  |
| Male | 1.05 | (0.15) | 0.90 | (0.09) | 1.14 | (0.10) | 1.50** | (0.23) | 1.15** | (0.22) | 0.04 | (0.03) |
| Age group |  |  |  |  |  |  |  |  |  |  |  |  |
| 16-29 years | Ref |  | Ref |  | Ref |  | Ref |  | Ref |  | Ref |  |
| 30-39 years | 2.32* | (0.79) | 0.71 | (0.16) | 1.04 | (0.21) | 0.67 | (0.24) | -0.74 | (0.59) | -0.06 | (0.07) |
| 40-49 years | 2.66** | (0.85) | 0.85 | (0.17) | 1.09 | (0.20) | 0.79 | (0.25) | -0.71 | (0.56) | -0.11 | (0.06) |
| 50-59 years | 2.54** | (0.80) | 0.88 | (0.17) | 1.18 | (0.21) | 0.52* | (0.16) | -0.81 | (0.55) | -0.11 | (0.06) |
| 60+ years | 2.58** | (0.81) | 0.60* | (0.12) | 0.99 | (0.18) | 0.53* | (0.17) | -0.70 | (0.56) | -0.08 | (0.06) |
| Urbanicity |  |  |  |  |  |  |  |  |  |  |  |  |
| Rural | Ref |  | Ref |  | Ref |  | Ref |  | Ref |  | Ref |  |
| Urban | 1.09 | (0.21) | 1.22 | (0.16) | 1.09 | (0.13) | 1.04 | (0.20) | 0.60** | (0.21) | 0.12** | (0.03) |
| Region |  |  |  |  |  |  |  |  |  |  |  |  |
| Papua, Maluku, NT | Ref |  | Ref |  | Ref |  | Ref |  | Ref |  | Ref |  |
| Java, Bali | 0.88 | (0.40) | 1.24 | (0.32) | 0.85 | (0.19) | 0.52* | (0.17) | -0.56 | (0.42) | 0.11* | (0.05) |
| Sumatra | 0.93 | (0.43) | 1.04 | (0.27) | 0.70 | (0.16) | 0.41** | (0.14) | -0.01 | (0.45) | 0.00 | (0.05) |
| Kalimantan | 0.96 | (0.46) | 0.75 | (0.21) | 0.86 | (0.22) | 0.60 | (0.24) | -0.39 | (0.49) | 0.04 | (0.06) |
| Sulawesi | 1.02 | (0.50) | 0.84 | (0.24) | 0.85 | (0.21) | 0.37** | (0.14) | 0.58 | (0.48) | 0.01 | (0.06) |
| Hospital ownership |  |  |  |  |  |  |  |  |  |  |  |  |
| Private | Ref |  | Ref |  | Ref |  | Ref |  | Ref |  | Ref |  |
| Government | 1.04 | (0.18) | 1.49** | (0.17) | 1.79** | (0.19) | 1.21 | (0.23) | 1.02** | (0.20) | -0.00 | (0.03) |
| Hospital level |  |  |  |  |  |  |  |  |  |  |  |  |
| Level A | Ref |  | Ref |  | Ref |  | Ref |  | Ref |  | Ref |  |
| Level B | 0.88 | (0.16) | 0.69** | (0.08) | 0.56** | (0.06) | 0.45** | (0.08) | -1.90** | (0.27) | -0.54** | (0.04) |
| Level C | 0.59* | (0.14) | 0.49** | (0.08) | 0.45** | (0.07) | 0.30** | (0.08) | -3.25** | (0.29) | -0.87** | (0.04) |
| Level D | 0.44 | (0.21) | 0.49 | (0.18) | 0.44** | (0.13) | 0.24** | (0.10) | -3.61** | (0.45) | -1.03** | (0.06) |
| Specialties | 0.66 | (0.25) | 0.89 | (0.17) | 1.55* | (0.31) | 0.90 | (0.28) | -0.94 | (0.55) | 0.18* | (0.07) |
|  |  |  |  |  |  |  |  |  |  |  |  |  |
| Constant | 0.06** | (0.04) | 0.35* | (0.14) | 0.84 | (0.31) | 0.51 | (0.29) | 7.65** | (0.80) | 16.12** | (0.11) |
| Observations | 9,885 |  | 9,885 |  | 9,885 |  | 9,885 |  | 9,885 |  | 9,885 |  |

Note: AOR=Adjusted Odds Ratio, SE=Standard errors, Ref=Reference, NT=Nusa Tenggara, PBI= Subsidized members, APBN=National government budget, APBD=Local government budget, Ln=Natural log. Transfusion included transfusion of packed cells and transfusion of platelets; Pooled logit regressions in Stata 15. Robust SE in parentheses. ** p<0.01, * p<0.05

**Appendix 5: Correlates of health services and outcomes among BPJS members with benign cancer, Indonesia 2017-2022**

|  | Biopsy | | Transfusion | | Severity | | Mortality | | LOS | | Claim cost (Ln) | |
| --- | --- | --- | --- | --- | --- | --- | --- | --- | --- | --- | --- | --- |
| Variables | AOR | (SE) | AOR | (SE) | AOR | (SE) | AOR | (SE) | Coef | (SE) | Coef | (SE) |
|  |  |  |  |  |  |  |  |  |  |  |  |  |
| Membership |  |  |  |  |  |  |  |  |  |  |  |  |
| PBI APBN | Ref |  | Ref |  | Ref |  | Ref |  | Ref |  | Ref |  |
| PBI APBD | 0.58* | (0.14) | 0.88 | (0.19) | 0.91 | (0.15) | 0.64 | (0.23) | 0.08 | (0.20) | -0.00 | (0.02) |
| Informal non-worker (BP) | 0.93 | (0.29) | 1.20 | (0.28) | 1.00 | (0.17) | 1.07 | (0.40) | 0.11 | (0.21) | 0.30** | (0.04) |
| Informal worker (PBPU) | 0.81 | (0.14) | 1.14 | (0.17) | 0.94 | (0.10) | 0.77 | (0.19) | -0.00 | (0.09) | 0.09** | (0.02) |
| Formal worker (PPU) | 0.79 | (0.14) | 0.71* | (0.11) | 0.64** | (0.07) | 0.69 | (0.18) | -0.29** | (0.09) | 0.25** | (0.01) |
| Data year |  |  |  |  |  |  |  |  |  |  |  |  |
| 2017 | Ref |  | Ref |  | Ref |  | Ref |  | Ref |  | Ref |  |
| 2018 | 0.88 | (0.17) | 1.18 | (0.18) | 1.17 | (0.14) | 1.27 | (0.33) | 0.11 | (0.11) | -0.01 | (0.02) |
| 2019 | 1.21 | (0.23) | 1.23 | (0.20) | 1.14 | (0.14) | 1.17 | (0.34) | -0.13 | (0.11) | -0.00 | (0.01) |
| 2020 | 1.27 | (0.27) | 1.21 | (0.23) | 1.36* | (0.19) | 2.82** | (0.94) | 0.26 | (0.16) | 0.03 | (0.02) |
| 2021 | 2.02** | (0.39) | 1.51* | (0.25) | 1.49** | (0.19) | 1.87* | (0.56) | 0.07 | (0.11) | 0.02 | (0.02) |
| 2022 | 1.34 | (0.26) | 1.25 | (0.23) | 1.10 | (0.15) | 0.84 | (0.24) | -0.18 | (0.10) | 0.00 | (0.02) |
| Sex |  |  |  |  |  |  |  |  |  |  |  |  |
| Female | Ref |  | Ref |  | Ref |  | Ref |  | Ref |  | Ref |  |
| Male | 0.51** | (0.07) | 0.62** | (0.07) | 0.84* | (0.07) | 1.33 | (0.26) | 0.07 | (0.09) | -0.14** | (0.01) |
| Age group |  |  |  |  |  |  |  |  |  |  |  |  |
| 16-29 years | Ref |  | Ref |  | Ref |  | Ref |  | Ref |  | Ref |  |
| 30-39 years | 0.54** | (0.09) | 1.94** | (0.37) | 2.11** | (0.31) | 1.13 | (0.52) | 0.23** | (0.08) | 0.02 | (0.02) |
| 40-49 years | 0.69* | (0.12) | 5.18** | (0.90) | 4.32** | (0.57) | 2.85** | (1.11) | 0.82** | (0.09) | 0.06** | (0.01) |
| 50-59 years | 0.85 | (0.18) | 4.16** | (0.91) | 3.91** | (0.60) | 3.74** | (1.53) | 0.88** | (0.13) | 0.01 | (0.02) |
| 60+ years | 0.90 | (0.18) | 5.47** | (1.15) | 5.06** | (0.80) | 4.71** | (1.96) | 0.98** | (0.12) | -0.03 | (0.02) |
| Urbanicity |  |  |  |  |  |  |  |  |  |  |  |  |
| Rural | Ref |  | Ref |  | Ref |  | Ref |  | Ref |  | Ref |  |
| Urban | 1.04 | (0.14) | 1.12 | (0.13) | 1.08 | (0.09) | 1.61* | (0.35) | 0.07 | (0.08) | 0.07** | (0.01) |
| Region |  |  |  |  |  |  |  |  |  |  |  |  |
| Papua, Maluku, NT | Ref |  | Ref |  | Ref |  | Ref |  | Ref |  | Ref |  |
| Java, Bali | 1.56 | (0.43) | 0.65* | (0.12) | 0.54** | (0.08) | 0.68 | (0.23) | -0.91** | (0.15) | -0.02 | (0.02) |
| Sumatra | 0.68 | (0.21) | 0.68* | (0.13) | 0.48** | (0.07) | 0.75 | (0.26) | -0.36* | (0.16) | -0.06** | (0.02) |
| Kalimantan | 1.12 | (0.37) | 0.84 | (0.18) | 0.61** | (0.11) | 0.95 | (0.36) | -0.80** | (0.17) | -0.04 | (0.03) |
| Sulawesi | 0.68 | (0.20) | 0.49** | (0.13) | 0.60** | (0.10) | 0.98 | (0.41) | -0.07 | (0.16) | -0.04 | (0.02) |
| Hospital ownership |  |  |  |  |  |  |  |  |  |  |  |  |
| Private | Ref |  | Ref |  | Ref |  | Ref |  | Ref |  | Ref |  |
| Government | 1.50** | (0.22) | 1.26 | (0.16) | 1.29** | (0.12) | 4.56** | (1.11) | 0.83** | (0.09) | -0.04** | (0.01) |
| Hospital level |  |  |  |  |  |  |  |  |  |  |  |  |
| Level A | Ref |  | Ref |  | Ref |  | Ref |  | Ref |  | Ref |  |
| Level B | 0.42** | (0.07) | 0.49** | (0.07) | 0.30** | (0.04) | 0.45** | (0.11) | -2.87** | (0.26) | -0.53** | (0.03) |
| Level C | 0.33** | (0.06) | 0.21** | (0.04) | 0.15** | (0.02) | 0.16** | (0.05) | -4.15** | (0.25) | -0.86** | (0.03) |
| Level D | 0.22** | (0.06) | 0.12** | (0.03) | 0.09** | (0.02) | 0.09** | (0.06) | -4.39** | (0.26) | -1.00** | (0.03) |
| Specialties | 0.41** | (0.14) | 0.72 | (0.26) | 0.51** | (0.13) | 1.89 | (0.93) | -2.42** | (0.73) | -0.45** | (0.08) |
|  |  |  |  |  |  |  |  |  |  |  |  |  |
| Constant | 0.16** | (0.07) | 0.10** | (0.04) | 0.40** | (0.12) | 0.01** | (0.00) | 6.25** | (0.36) | 16.14** | (0.05) |
| Observations | 25,019 |  | 25,019 |  | 25,019 |  | 25,019 |  | 25,019 |  | 25,019 |  |

Note: AOR=Adjusted Odds Ratio, SE=Standard errors, Ref=Reference, NT=Nusa Tenggara, PBI= Subsidized members, APBN=National government budget, APBD=Local government budget, Ln=Natural log. Transfusion included transfusion of packed cells and transfusion of platelets; Pooled logit regressions in Stata 15. Robust SE in parentheses. ** p<0.01, * p<0.05
