# Supplementary material for: Differences in Hemodialysis Claim Patterns Across Membership Types Among Patients With Renal Failure Based on National Health Insurance Data From 2017 to 2022: Cross-Sectional Analysis
Source: JMIR Public Health Surveill. 2025 Nov 3;11:e73731. doi: 10.2196/73731 (PMC12624297; doi:10.2196/73731)
Supplement: Multimedia Appendix 2 [file publichealth_v11i1e73731_app2.docx]

**Appendix 1. The flowchart outlining the sample selection process**

Sampel klaim

| 6,897,148 | |  | Total claims 2017-2022 |
| --- | --- | --- | --- |
|  |  |  | 6,858,361 claims not ICD-10 N17 - N19 removed |
|  |  |  |  |
| 38,787 | |  |  |
|  |  |  | 404 claims of samples < 18 years old removed |
|  |  |  |  |
| 38,383 | |  | Final sample |

**Appendix 2. Total Number of Claims and Unique Patients by Year in the Sample Dataset, Indonesia (2017–2022)**

| **Year** | **Total Claims** | **Unique Patients** |
| --- | --- | --- |
| 2017 | 5,262 | 974 |
| 2018 | 6,423 | 1,246 |
| 2019 | 6,683 | 1,411 |
| 2020 | 6,366 | 1,375 |
| 2021 | 6,390 | 1,321 |
| 2022 | 7,259 | 1,589 |
| Total | 38,383 | 7,916 |

Note: Only a small proportion of patients had repeated claims across years. For example, just 30.7% of patients identified in 2017 reappeared in 2018, and this proportion declined further in subsequent years. This limited overlap supports the use of a cross-sectional, claim-level approach rather than a panel analysis. See Appendix 2.
